# Role of Receptor Tyrosine Kinase Signaling in Renal Fibrosis

**DOI:** 10.3390/ijms17060972

**Published:** 2016-06-20

**Authors:** Feng Liu, Shougang Zhuang

**Affiliations:** 1 Department of Nephrology, Shanghai East Hospital, Tongji University School of Medicine, Shanghai 200120, China; liufeng0113@126.com; 2 Department of Medicine, Rhode Island Hospital, Alpert Medical School of Brown University, Providence, RI 02903, USA

**Keywords:** receptor tyrosine kinases, platelet-derived growth factors receptor, fibroblast growth factor receptor, vascular endothelial growth factors receptor, epidermal growth factor receptor, insulin-like growth factor receptor, discoidin domain receptors, growth arrest-specific gene, tyrosine kinase inhibitors, renal fibrosis

## Abstract

Renal fibrosis can be induced in different renal diseases, but ultimately progresses to end stage renal disease. Although the pathophysiologic process of renal fibrosis have not been fully elucidated, it is characterized by glomerulosclerosis and/or tubular interstitial fibrosis, and is believed to be caused by the proliferation of renal inherent cells, including glomerular epithelial cells, mesangial cells, and endothelial cells, along with defective kidney repair, renal interstitial fibroblasts activation, and extracellular matrix deposition. Receptor tyrosine kinases (RTKs) regulate a variety of cell physiological processes, including metabolism, growth, differentiation, and survival. Many studies from *in vitro* and animal models have provided evidence that RTKs play important roles in the pathogenic process of renal fibrosis. It is also showed that tyrosine kinases inhibitors (TKIs) have anti-fibrotic effects in basic research and clinical trials. In this review, we summarize the evidence for involvement of specific RTKs in renal fibrosis process and the employment of TKIs as a therapeutic approach for renal fibrosis.

## 1. Introduction

In the general population, the prevalence of chronic kidney disease (CKD) is more than 10%. Many patients with CKD will eventually progress to end-stage of renal disease (ESRD), which necessitates replacement therapy. Progressive renal function loss in CKD patients is associated with the development of glomerulosclerosis and/or progressive tubular interstitial fibrosis (TIF). Although both fibrotic lesions are important, the reduction in the glomerular filtration rate (GFR) has been correlated better with tubular interstitial lesions than with glomerulosclerosis [1]. Despite the fact that there are many different causes, renal fibrosis is a consistent outcome for CKD patients. Therefore, understanding how to prevent renal fibrosis and delay the progression of renal dysfunction is critical for improving the prognosis of patients with CKD [1]. Many pharmaceutical companies have been committed to the study of how to delay the onset and development of renal fibrosis, but the translation of new treatment options to clinical applications are still lacking good performance [2].

Renal fibrosis is characterized by the proliferation and transdifferentiation of renal interstitial fibroblasts to myofibroblasts, and the deposition of extracellular matrix. This process is involved in the activation of growth factor receptors, including receptor tyrosine kinases (RTKs) and their downstream signaling pathways. In this review, we discuss the role of specific RTKs in renal diseases, with a particular focus on their roles in progressive renal diseases, and the implications of tyrosine kinases inhibitors (TKIs) in renal fibrosis.

## 2. Receptor Tyrosine Kinases

Tyrosine kinases have many regulatory functions in cell physiological processes, including metabolism, growth, differentiation, and apoptosis. RTKs and non-receptor tyrosine kinases (nRTKs) are the two subtypes of tyrosine kinases. RTKs are membrane receptors, which include extracellular, transmembrane and intracellular domains, while nRTKs, including Src, c-Abl, and c-kit, lack extracellular and transmembrane domains. RTKs are classified by a variety of superfamilies, including platelet-derived growth factor receptors (PDGFR), vascular endothelial growth factor receptors (VEGFR), fibroblast growth factor receptors (FGFR), epidermal growth factor receptors (EGFR), insulin-like growth factor receptors (IGFR), discoidin domain receptors (DDRs), Axl receptor tyrosine kinase (AXL) [3]. The classification of RTKs superfamilies and their ligands are shown in Table 1. Currently, 20 known RTKs have been identified in humans [3]. Given the central role of RTKs in cell signaling, pathologic activation or blocking of tyrosine kinases can drive or inhibit the development and progression of carcinogenesis, vascular remodeling, and fibrogenesis of several tissues [4,5].

All RTKs have a common molecular architecture and are composed of three domains: ligand-binding domains in the extracellular region, a single transmembrane helix, and a cytoplasmic region in the intracellular regions, which contains the tyrosine kinase and C-terminal domains [3]. RTKs binding to the specific ligand, activate, dimerize and induce tyrosine residue autophosphorylation, which is followed by the activation of intrinsic protein tyrosine kinase activity. Collectively, specific intracellular adaptor proteins like phospholipase C γ (PLCγ), growth factor receptor-bound protein 2 (Grb2), Src, and Shc are activated by these docking phosphotyrosines, and followed by activation of intracellular signal transduction pathways, such as phosphatidylinositol 3-kinase (PI3K)/Akt, Ras/extracellular signal-regulated (Erk1/2) mitogen-activated protein (MAP) kinases or protein kinase C (PKC) [3,6].These downstream signaling pathways are involved in cell differentiation, apoptosis, migration and reassembly of the cytoskeleton [3].

Many studies have shown that appropriate RTK monoclonal antibodies and inhibitors can effectively inhibit tumor cell differentiation, apoptosis and angiogenesis; additionally, they are shown to contribute to the prevention and treatment of pulmonary, heart, liver and dermal fibrosis [7]. Therefore, administration of TKIs may aid in the prevention and treatment of renal fibrosis. Since RTKs (PDGFR, VEGFR, EGFR, IGFR, DDR and Axl) play a key role in renal fibrosis [8], understanding the mechanism by which RTKs mediate renal fibrosis is important for intervention in renal fibrosis.

## 3. Platelet-Derived Growth Factor (PDGF) and Platelet-Derived Growth Factor Receptors (PDGFR)

The platelet-derived growth factor (PDGF) family consists of four isoforms (PDGF-A, -B, -C, -D) and two receptor subtypes (PDGFR-α and-β). PDGFR-α and -β are constitutively expressed in most renal inherent cells, including mesangial cells, interstitial fibroblasts and vascular smooth muscle cells [9,10,11,12]. They are also functionally involved in kidney development, mesangial cell proliferation and fibrogenesis [13]. There is accumulating evidence indicating that PDGF families are the best-characterized RTKs in normal and diseased kidneys, especially in the development of renal fibrosis due to multiple underlying pathogens. PDGF/PDGFR is involved in a variety of pathophysiologic events in renal cells, such as cell proliferation and migration, extracellular matrix (ECM) accumulation, pro- and anti-inflammatory mediators production, tissue permeability and hemodynamics [11].

Numerous studies have proved the important role of PDGF/PDGFR in the pathogenesis of renal fibrosis. PDGF-B, instead of PDGF-A, injections induced renal interstitial fibroblasts proliferation and differentiation into myofibroblasts followed by renal interstitial fibrosis [14]. Mice with a PDGFR-α-activating mutation resulted in multiple organ fibrosis, including in the kidneys [15]. Eitner *et al.* [15] also showed that the fibrosis process in murine obstructive nephropathy was inhibited by both genetic PDGF-C deficiency and neutralizing antibodies to PDGF-C. In the murine model of unilateral ureteral obstruction (UUO), we found that suramin, an inhibitor of multiple RTKs, including PDGFR and EGFR, attenuates glomerular and vascular injury and reduces inflammatory responses [16]. In addition, in rat anti-Thy 1.1 mesangioproliferative glomerulonephritis, glomerular and tubulointerstitial scarring, as well as the subsequent development of renal failure, can be prevented by PDGF-D-specific monoclonal antibody [17,18]. In contrast, Trapidil, a nonspecific PDGF antagonist, worsened the clinical and histological outcome in rabbits with nephrotoxic nephritis, and in rats with renal ischemia/reperfusion injury [19,20]. Animal and clinical studies have also shown that imatinib, which can inhibit selective RTKS, including PDGFRα, ameliorated mesangioproliferative glomerulonephritis in rats, glomerular and tubulointerstitial lesions in experimental diabetic nephropathy (DN). Furthermore, Imatinib effectively reduced glomerular hypercellularity, tubulointerstitial fibrosis, activation of myofibroblasts in mice with lupus nephritis(LN) and prevented chronic allograft nephropathy (CAN) [12]. Another TKI for PDGFR, AG 1295, can also prevent the development of TIF in rats with UUO [21]. Therefore, inhibition of PDGFR will offer a therapeutic strategy for the prevention of the progression of glomerulosclerosis and tubulointerstitial fibrosis.

## 4. The Vascular Endothelial Growth Factor (VEGF) and Vascular Endothelial Growth Factor Receptors (VEGFR)

The vascular endothelial growth factor (VEGF) family members include VEGF-A, -B, -C, -D, -E, and placenta growth factor [22]. VEGF is an endothelial-specific growth factor that promotes endothelial cells’ (ECs) proliferation, differentiation and survival, by preventing endothelial apoptosis. Increased expression of interstitial plasminogen activator inhibitor-1(PAI-1) and collagenase, and VEGF are involved in mediating endothelium-dependent vasodilatation, microvascular hyperpermeability and interstitial matrix remodeling. Furthermore, VEGF participates in inflammation response by promoting monocyte chemotaxis and adhesion molecules expression [23].The receptors for VEGF have two subtypes, VEGFR-1 and VEGFR-2, which are high-affinity RTKs [23]. Hypoxia and other growth factors and cytokines, such as EGF, transforming growth factor β (TGF-β), PDGF, IGF-1, angiotensin II (AngII), interleukin-1 (IL-1), and IL-6 are the main stimuli for VEGF. VEGF expression and/or production may also be induced by other agents, such as prostaglandins, mechanical stress, hyperglycemia, advanced glycation end products (AGEs), protein kinase C (PKC), and reactive oxygen species (ROS) [23]. Most of these stimuli play important roles in the pathogenesis of renal fibrosis. In a negative feedback regulatory mechanism, nitric oxide (NO) can down-regulate VEGF expression and function [24].

VEGF are prominently expressed in glomerular podocytes and tubular epithelial cells, while VEGFR are mainly expressed in glomerular and peritubular endothelial cells [22].It has been reported that VEGF/VEGFR are involved in the induction and maintenance of the fenestrae, differentiation and proliferation, interstitial matrix remodeling and calcium homeostasis in glomerular capillary epithelial cells, as well as survival in human podocytes [25,26]. Human data also showed that plasma levels of VEGF in uremic patients were higher than that in control subjects [27]. In the animal model of DN, VEGF and its receptor expression increased significantly, and demonstrated that they are required for stimulation of glomerular cell proliferation and hardening [28]. VEGF and its receptors are involved in the glomerulus and tubule interstitial repair in the renal injury by thrombotic microangiopathy (TMA)/hemolytic uremic syndrome (HUS) [29,30], and cyclosporine nephrotoxicity [31]. In an experimental TMA model, subcutanous administration of VEGF_121_ resulted in improved renal microvasculature, renal function and TIF lesion severity [29]. It was also suggested that VEGF_121_-treated rats had higher urinary nitrites and nitrates excretion which was due to the angiogenic effects of VEGF mediated by NO [29].In a more severe renal TMA models with acute massive renal infarction that were manifested by glomerular endothelial cell apoptosis, microvascular endothelium, cortical and medullary necrosis, and severe lesions could be prevented by VEGF administration [30]. In a rat model of chronic cyclosporine nephrotoxicity, VEGF, as well as VEGFR-1 and VEGFR-2 expression increased as early as seven days after exposure [32,33,34]. They were mainly expressed in proximal and distal tubular cells, and occasionally in glomerular podocytes [33]. By decreasing VEGF and VEGFR-2 expression, enalapril or losartan could improve the tubulointerstitial fibrosis and afferent arteriolopathy, which may be due to the direct regulating effect of AngII on VEGFR [33]. In mutation diabetic mice (db/db), administration of anti-VEGF-antibodies also attenuated kidney weight, glomerular volume, and urinary albumin excretion; moreover, it abolished the increase in basement membrane thickness, reduced the mesangial matrix expansion, and improved creatinine clearance [35]. A monoclonal neutralizing VEGF-antibody could improve high protein-induced glomerular hypertrophy without influencing kidney and body weight [36]. It is suggested that VEGF is the downstream mediator of IGF-1 that involves in high protein-induced glomerular growth [36]. In the nephron reduction process, VEGF is related to compensatory glomerular, renal tubular hypertrophy and endothelial cell proliferation. However, several studies showed that down-regulation of VEGF could cause severe tubulointerstitial lesions and glomerulosclerosis [37,38]. As a stimuli for VEGF, AngII could increase its expression in mesangial cells, and decrease its expression in renal tubular epithelial cells [38,39]. One study demonstrates that administration of a selective and potent inhibitor of VEGF to rat with crescentic glomerulonephritis, one type of VEGFR, soluble Fms-like tyrosine kinase receptor 1 (sFlt-1) accelerates proteinuria with massive ascites, glomerulosclerosis and interstitial fibrosis, which is also associated with a loss of nephrin and endothelium [40]. Meanwhile, since VEGF and VEGFR are involved in the regulation of podocyte function, they play key roles in the pathogenesis of glomerulonephritis, which is demonstrated in minimal change nephropathy (MCD), membranous glomerulonephritis, membranoproliferative glomerulonephritis, mesangioproliferative glomerulonephritis, focal segmental glomerulosclerosis (FSGS), and IgA nephropathy [22]. Depressed VEGF synthesis induced by podocyte injury may contribute to endothelial cell loss and accelerate the progress of glomerulosclerosis. In the mouse model of UUO, VEGF treatment significantly reduced TIF by blocking the expression of α-smooth muscle actin (α-SMA), TGF-β1, and connective tissue growth factor (CTGF), and increasing the expression of E-cadherin and bone morphogenetic protein-7 (BMP-7), which indicated that VEGF may ameliorate TIF at an early stage in UUO mice [41]. As an anti-VEGF agent, bevacizumab (avastin) has been proved to inhibit fibrotic process in liver fibrosis, retinal or choroidal neovascularization and peritoneal sclerosis. In hepatic stellate cells, bevacizumab attenuated the development of hepatic fibrosis and improved liver function by downregulating the expression of α-SMA and TGF-β1 [42]. In an experimental peritoneal sclerosis model, bevacizumab could also reduce fibrotic process. In retinal pigment epithelial (RPE) cells, bevacizumab increased matrix metalloproteinases-2 (MMP-2) and MMP-9, but decreased VEGF-A and VEGFR-1 expression [43]. However, in human umbilical vein endothelial cells (HUVECs) *in vitro*, Zhang *et al.* found that bevacizumab at clinical doses exerts pro-fibrotic effects by upregulating the expression of CTGF, bFGF, and MMP-2 [44]. Hence, the effect of bevacizumab on renal fibrosis is still unknown, which is necessary for further study.

In summary, all of the above results suggest that VEGF and its receptors are involved in regulating normal renal glomerular permeability, podocyte function, and proliferation of renal inherent cells. However, the role of VEGF and its receptor in normal conditions may be different, or even opposite, from its role in renal injury. Therefore, it is necessary to select effective means to target VEGF/VEGFR signaling for the prevention and treatment of renal fibrosis.

## 5. Fibroblast Growth Factor (FGF) and Fibroblast Growth Factor Receptors (FGFR)

The fibroblast growth factor (FGF) family includes at least 23 heparin-binding proteins, with different biologic functions in developmental and repair processes of tissues [45]. FGFs act through binding to high-affinity FGFR, encoded by four genes that, through alternative mRNA splicing, can generate seven functional FGFR variants [46]. During embryonic development, FGF/FGFR are involved inmesoderm organization, body axis and neural axis formation, and tissue/organ induction [47,48]. Of all the FGF families, FGF2 (basic FGF (bFGF)) and its receptor, FGFR1, have been proven to be involved in the pathogenesis of renal disease by inducing fibrosis and contributing to renal damage in immune-mediated injury [49,50].

FGF and FGFR are widely distributed in the glomerular mesangium, glomerular endothelium, glomerular blood vessels, the interstitium, and within distal tubular epithelial and vascular smooth muscle cells (VSMC) [51]. In the normal physiological state, FGF and FGFR are involved in regulating mesangial cells, epithelial and vascular endothelial cell proliferation, differentiation and matrix synthesis through autocrine and paracrine pathways [51]. In pathological conditions, FGF and FGFR are involved in inflammatory processes of glomerularnephritis, glomerular sclerosis, and renal interstitial fibrosis through the regulation of transdifferentiation of tubular epithelial cells and interstitial fibroblasts [49]. In experimental animal models of membranous nephropathy, mesangioproliferative glomerulonephritis, IgA glomerulonephritis, CAN, and FGF-2/FGFR1 expression increased several times in cell proliferation regions, which are closely related to the degree of podocyte damage and proteinuria [52,53]. Similarly, subcutaneous FGF-2 injections to rats for 8 to 13 weeks, resulted in pathologic change manifested by FSGS, with an increase in the peritubular interstitium [54]. In a transgenic mouse model of human immunodeficiency virus (HIV)-associated nephropathy, Ray *et al.* [55] found that the expression of FGF-2 increased in interstitial regions and co-localized with ECM. In human kidneys with tubulointerstitial scarring, Strutz *et al.* [56] also found that the expression of FGF-2 protein was increased, correlating with the degree of interstitial fibrosis. Results also showed that FGF-2 mRNA expression was robustly increased in interstitial and tubular cells in end-stage kidneys, as well as FGF-2-induced expression of α-SMA, with no significant effect on the synthesis of collagen type I and fibronectin. In the cultured transformed medullary fibroblast line, primary cortical kidney, and skin fibroblasts isolated from human biopsies, Strutz *et al.* [56] demonstrated that administration of a neutralizing antibody to FGF-2 could completely block fibroblast proliferation induced by TGF-β1. The same effect was shown with the use of another TKI, AG1296, which could block FGFR activity. However, neutralizing antibodies of EGF and PDGF did not have this effect [56]. This suggests that FGF-2 is the downstream factor of TGF-β signaling. Although the role of FGF-1 (acidic FGF (aFGF)) in renal disease is less studied, FGF-1 may be involved in inflammatory diseases, such as rheumatoid arthritis. In addition, increased FGF-1/FGFR expression was observed in the fibrotic areas of renal biopsy specimens from patients with LN and acute interstitial nephritis. This supports the possibility that FGF-1 may contribute to infiltrating inflammatory cells and associated fibrosis, and plays an autocrine and paracrine role in tubulointerstitial remodeling after injury.

## 6. Epidermal Growth Factor (EGF) and Epidermal Growth Factor Receptor (EGFR)

Epidermal growth factor receptor (EGFR) is the inaugural member of the ErbB family of RTKs. Its activation initiates several signaling pathways, including the extracellular signal-regulated kinase (ERK), PI3K/Akt and jun kinase (JNK) pathways, which are involved in regulating cell proliferation, survival, differentiation, migration, inflammation, and matrix homeostasis [57]. There are several ligands for EGFR, such as epidermal growth factor (EGF), transforming growth factor-α (TGF-α), heparin-binding EGF (HB-EGF), amphiregulin (AREG), betacellulin (BTC), and epiregulin (EREG). All of them are synthesized as transmembrane precursors and then cleaved by MMPs and/or the ADAM (a disintegrin and metalloproteinase) family to release mature ligands [58]. EGFR is widely expressed in most renal inherent cells, including mesangial cells, glomerular epithelial cells and tubular interstitial fibroblasts, and plays an important role in the development of the kidney [59].

Several studies indicated that EGFR is a major determinant in the initiation and development of renal injury. Addition of EGFR ligands to a cultured renal tubular cells promotes several biological responses, including cell proliferation, mesenchymal-epithelial transdifferentiation and collagen production [59]. It has been reported that activation of EGFR may be involved in the evolution of multiple renal diseases. For example, expression of a dominant negative isoform of EGFR in proximal tubules inhibits tubular cell proliferation and interstitial collagen accumulation, leading to reduced renal lesions after nephron reduction [59]. EGFR has been confirmed to play a key role in tubular cyst formation and cell proliferation in polycystic kidney diseases [60], and in renal fibrosis in an experimental hypertension model [61]. In both mice and humans with rapidly progressive glomerulonephritis (RPGN), Bollée *et al.* [62] found *de novo* induction of HB-EGF in intrinsic glomerular epithelial cells (podocytes) and its induction increased phosphorylation of EGFR in mice with RPGN [62]. Meanwhile, in HB-EGF–deficient mice, EGFR phosphorylation in glomeruli was absent and the lesions severity of RPGN was improved. Autocrine HB-EGF induces a phenotypic switch in mice podocytes *in vitro*, and conditional deletion of the EGFR gene from podocytes could alleviate the severity of RPGN [62].

**Figure 1:**
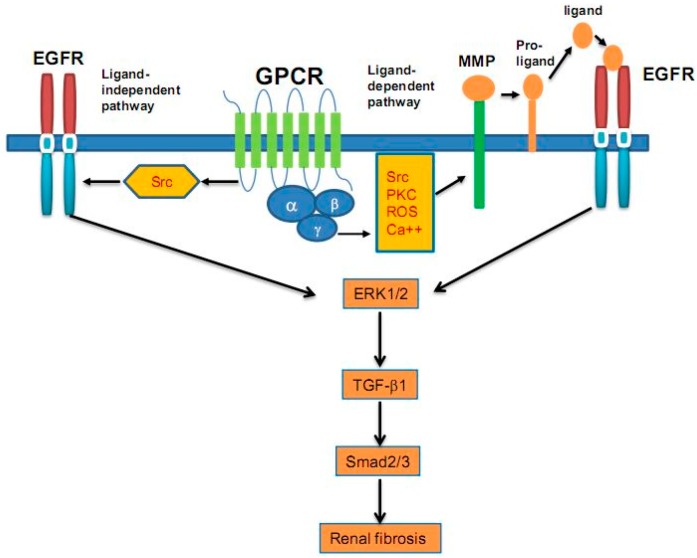
Signaling mechanisms of epidermal growth factor receptor (EGFR) transactivation in renal fibrosis. Upon stimulation with G protein-coupled receptor (GPCR) ligands (*i.e.*, angiotension II), some intracellular events. Including activation of Src and protein kinase C (PKC), production of reactive oxygen species (ROS) and influx of calcium are initiated. Src can directly induce EGFR phosphorylation/activation via a ligand-independent mechanism. Src and other intracellular signaling molecules can also induce EGFR transactivation via a ligand dependent mechanism involving activation of MMPs and subsequent cleavage of proEGFR ligands to release their soluble forms. These soluble ligands bind to EGFR and induce its dimerization and activation, which, in turn, sequentially activates the ERK1/2, production of TGF-β1 and activation of TGF-β signaling pathway.

TGF-β is recognized as a central mediator of organ fibrosis, and AngII-mediated fibrogenesis is mediated by TGF-β signaling. Studies have indicated that EGFR is involved in the AngII and TGF-β-mediated renal fibrosis. In a mouse model, overexpressing a dominant negative isoform of EGFR, Lautrette *et al.*, showed that AngII-dependent EGFR transactivation is indispensable for renal deterioration [63]. Chen *et al.* [64] demonstrated that activation of ERK signaling, induced by EGFR, was involved in mediating TGF-β expression in renal fibrosis. TGF-β expression can be regulated by sustained EGFR-dependent signaling, which is activated by the ROS-dependent phosphorylation of Src due to the Ang II receptor activation [63]. Erlotinib, one inhibitor for EGFR, could significantly decreased TGF-β-mediated fibrogenesis [64]. It was also found that EGFR phosphorylation in wild type mice was higher than that in waved-2 mice, in which EGFR tyrosine kinase activity was reduced by 90%, and treatment with suramin, an inhibitor of multiple RTKs, including EGFR, could improve renal fibrosis by inhibiting α-SMA, TGF-β, Smad3, STAT3, ERK1/2 expression [16]. In the 5/6 nephrectomy renal fibrosis animal model, inhibition of EGFR and other RTKs by suramin could reduce fibrosis factor expression, and improve inflammatory cells infiltration [65]. In an ischemic/reperfusion (I/R)-induced mice model, we also showed that severe I/R injury induced a sustained activation of EGFR, which is required for renal regenerative responses (tubular cell dedifferentiation and proliferation) in the early phase, but is, in turn, a critical mediator of renal fibrogenesis in the later phase of acute kidney injury (AKI) [66]. This suggests that EGFR exerts dual roles in the course of severe AKI, and that sustained EGFR activation is a critical molecular event that leads to development of TIF. As a ligand for EGFR, TGF-α contributes to the development of CKD. In FVB/N mice, Laouari *et al.* [67] revealed that TGF-α protein levels significantly increased after nephron reduction, and this increase preceded the progress of renal lesions. These renal lesions could be blocked by one inhibitor for EGFR (Iressa) [67]. Therefore, EGFR ligands and/or their upstream factors involve in renal fibrogenesis, and blockade of the EGFR signaling pathway could be a potential therapeutic method for treating renal fibrosis. Signaling mechanisms of EGFR transactivation in renal fibrosis are shown in Figure 1.

TGF-β1 has been generally considered a potent pro-fibrotic mediator by regulating epithelial-mesenchymal transition (EMT), ECM production and inflammation in the process of renal fibrosis [68]. Pirfenidone, a new anti-TGF-β agent, exhibits both anti-fibrotic and anti-inflammatory effects. In experimental models, pirfenidonereduces fibroblast proliferation, TGF-β expression and the expression of inflammatory mediators, such as TNF-α and IL-1 [69]. Therefore, TGF-β and downstream factors are considered to be the ideal therapeutic targets for the treatment of organ fibrosis. However, the effect of pharmacological blockade of TGF-β in clinical practice for humans is still controversial. In streptozotocin (STZ)-induced and Leprdb/db diabetic mice, neutralizing anti-TGF-β antibody could significantly reduce TGF-β1 levels and inhibit glomerular mesangial matrix expansion [70]. However, the recent multicenter phase II trial sponsored by Eli Lilly, using LY2382770, a humanized neutralizing monoclonal antibody against TGF-β1 for the treatment of DN had to be prematurely terminated due to futile inefficacy [68]. A 157 phase II multicenter, double-blind, randomized study of fresolimumab, a human monoclonal antibody that neutralizes all three isoforms (TGF-β1, 2 and 3), in patients with steroid-resistant primary FSGS was recently completed but yielded no convincing study results [68].

Drugs targeting AngII action or production, such as angiotensin-converting enzyme (ACE) inhibitors and AngII receptor blockers (ARB), have a protective effect in diabetic and non-diabetic nephropathies, since they can reduce proteinuria and the rate of loss of renal function, and reduce cardiovascular risk in CKD patients [71]. However, the magnitude of this effect has been questioned, particularly in mild, proteinuric nephropathies. In addition, there is cross-talk among AngII receptors and RTKs [63]. Both Ca^2+^-dependent (Src) and Ca^2+^-independent AngII induced EGF transactivation pathways exist in cardiac fibroblasts, and for PDGFR isoforms, but PDGFR-β transactivation is through AngII, independent of Ca^2+^ [2]. In cardiac fibroblasts and VSMC, AngII-induced ERK1/2 phosphorylation occurs via transactivation of EGFR and PDGFR-β [63], but in mesangial cells, ERK1/2 activation only exists in the process of PDGFR-β by AngII [72]. TGF-β and AngII have been proved to be pro-fibrotic agents in the kidney. The precise molecular mechanisms of cross-talk and/or transactivation among RTKs with TGF-β and AngII need to be further investigated.

## 7. Insulin-Like Growth Factors (IGF) and Insulin-Like Growth Factor Receptors (IGFR)

Insulin-like growth factors (IGF-1 and IGF-2) play a critical role in normal growth and development [73]. The IGF family is structurally related to proinsulin, and regulates cell proliferation, differentiation, and survival, as well as insulin-like metabolic effects. IGFs are expressed in many cell types and have autocrine, endocrine, and paracrine actions. The IGF superfamily includes receptors for IGF-1, IGF-2/mannose-6-phosphate, insulin, and a family of six high-affinity IGF-binding proteins (IGFBPs) [74]. Most actions of IGFs are mediated by the tyrosine kinase IGF-1 receptors (IGFR), whereas, the IGF-2/mannose-6-phosphate receptors predominantly act as clearance receptors for IGF-2. IGFBPs primarily inhibit IGF actions, although they may enhance them in some circumstances. Many growth hormone (GH) actions are mediated by IGF-1 and IGFR.

IGF-1 expresses in mesangial cells, podocytes and tubular cells. The GH/IGF-1 system plays a critical role in normal kidney development and function [75]. IGFs can regulate renal plasma flow and glomerular filtration rate (GFR) by increasing the ultrafiltration coefficient and decreasing efferent arteriolar resistance by IGF-1-stimulated NO production and cyclooxygenase [76,77]. Both GH and IGF-1 increase sodium reabsorption in the distal nephron, and have roles in phosphate and calcium homeostasis [78]. Following unilateral nephrectomy, the remaining kidney undergoes compensatory growth, which is dependent on GH/IGF-1 [78]. IGF-1 is also a potent mesangial cell mitogen that stimulates cell migration and the production of ECM proteins while inhibiting apoptosis induced by high glucose levels [79]. The GH/IGF-1 system has protective and deleterious effects on podocytes. Podocyte depletion is associated with abnormal glomerular IGF expression. However, the podocyte produces IGF-1 via the IGFR and PI3K signaling pathways [80,81,82]. The actions of IGF-2 were found to be mediated by IGFR and were observed to be crucial for podocyte cell survival and the integrity of the glomerular filtration barrier [83].

Disordered regulation of the IGF system has been implicated in various CKDs, including DN, polycystic kidneys and proteinuric CKD [76,84]. IGF-1 accumulates in kidneys prior to the onset of hypertrophy in diabetic rodent models, such as the STZ-induced diabetic rats, mice and non-obese diabetic (NOD) mice, and IGF-1 receptor levels are also increased in animal models of DN [85]. In patients with DN, urinary IGFBP-3 protease activity correlates with the degree of albuminuria [86]. Targeting the GH/IGF results in renoprotective effects [87]. Serum IGF-1 levels prior to commencing dialysis therapy were correlated with measures of nutritional status and bone metabolism in patients with ESRD, and low IGF-1 levels were related with increased mortality in dialysis patients [88]. IGF-1 is increased in regenerating proximal tubule cells after injury in rats. IGF-1 treatment accelerates recovery in animal models of AKI, but clinical trials had controversial results [78]. IGFBP-3 and IGFBP-1 levels are increased in patients with FSGS, but not in patients with MCD [89]. IGFBP-1 levels are also elevated in the kidneys in a mouse model of experimental IgA glomerulonephritis and in serum of patients with IgA glomerulonephritis. This correlates with mesangial cell proliferation and other markers of kidney damage [90]. IGFs can stimulate ECM protein accumulation in renal inherent cells, induce EMT in collecting duct epithelial cells [91], and increase fibronectin and collagen expression in rat mesangial cells and proximal tubular cells [92]. IGF-1 and TGF-β have a synergistic effect on ECM protein accumulation [93]. In addition, the activation of CTGF, a downstream mediator for TGF-β, is dependent on IGF-1 [94]. In the 5/6 subtotal nephrectomy (SNx) rat, IGF-1 expression was correlated with damage in the tubulointerstitial space and injured distal tubular cells [95]. However, IGF-1 can reduce renal interstitial collagen deposition, tubular vimentin expression and apoptosis, indicating an inhibiting effect on fibrotic processes [96]. In SNx rats, Simon *et al.* [95] did not find an IGFR antagonist, JB3 could prevent the development of renal fibrosis. Therefore, IGF-1 and its receptor play a limited role in renal fibrogenesis, but have significant effects on the regulation of renal growth.

## 8. Discoidin Domain Receptor 1 (DDR1) and Discoidin Domain Receptor 2 (DDR2)

As candidate effectors in tissue injury and fibrosis, the discoidin domain receptors (DDRs), including DDR1 and DDR2, are cardinal members of a RTKs subfamily, activated by collagen I–VI and VIII [97]. Different from other RTKs, DDRs have a longer juxtamembrane domain and a unique activation pattern that takes several hours after an initial stimulation [97]. In normal kidney, DDR1 is expressed in basolateral membranes of nephron segments, from the connecting tubule to the renal papilla. DDR2 is expressed in apical membranes of select nephron segments, from the loop of Henle to the macula densa. In the remnant kidney, DDR1 and DDR2 mRNA and protein expression are upregulated within the glomeruli [97].

DDR1 and DDR2 modulate inflammatory recruitment, ECM deposition and fibrosis. Like most RTKs, MAP kinase and PI3K signaling are the downstream pathway of DDR-1. In a hypertensive model of renal disease, induced by AngII, DDR1 was overexpressed in glomeruli and renal vessels [98]. In a UUO model, DDR1 was overexpressed in interstitial cells, especially in macrophages [99]. In DDR-1 knockout (KO) mice, collagen I and IV deposition in renal tissue, T lymphocyte and macrophage infiltration decreased significantly [98]. The migratory activity of inflammatory cells isolated from DDR1 KO mice were significantly downregulated [99] while renal function, histological lesions and mortality were improved [100]. In an animal model induced by antibodies against the glomerular basement membrane, DDR1 was involved in the injury of crescentic glomerulonephritis. Imatinib and nilotinib, the inhibitors for multiple RTKs, including DDR1, can regulate the immune response and prevent the development of fibrogenesis [101]. However, their side effects limit their evaluation in clinical trials.

## 9. Growth Arrest-Specific Gene 6 (Gas6) and Axl Receptor

The Axl subfamily is a founder of a unique TAM (Tyro3, Axl and Mertk) family of RTKs, which is composed of Axl, Sky and Mer. Growth arrest-specific gene 6 (Gas6), as a growth factor-like molecule, which belongs to the family of vitamin K-dependent coagulation proteins, has the highest affinity for Axl, and is often called the Gas6/Axl pathway [102]. Gas6/Axl signaling regulates various cellular functions, such as survival, proliferation, adhesion, migration, blood clot stabilization, and inflammatory cytokine release [102]. Within the kidney, Axl and Gas6 are expressed in VSMCs, glomerular mesangial cells and tubular cells [103].

The basal expression of Gas6/Axl is low in normal kidney. However, its expression can be upregulated in several murine models of CKD including: glomerulonephritis induced by Thy1.1 antibody, nephrotoxic nephritis (NS), STZ-induced DN and a podocyte ablation model of glomerular albuminuria [103]. Patients with CKD had elevated plasma Gas6 levels [104]. In diabetic rats and mice induced by STZ, the expression of Gas6/Axl and its downstream signaling of Akt/mTOR increased in the glomerulus, followed by mesangial and glomerular hypertrophy [105,106]. In VSMCs, Gas6/Axl was involved in the increase of cell survival in the presence of low glucose, and cell migration in the presence of high glucose (HG) [107]. Patients with inflammatory kidney disease [103] and IgA glomerulonephritis [108] had elevated plasma Gas6/Axl levels. In a murine sub-total nephrectomy and high phosphate diet model, the levels of plasma Gas6, renal Axl and Akt were significantly elevated, and tubulointerstitial apoptosis, uraemia and mortality increased, resulting from Axl loss [103]. In acute rejection, acute tubular necrosis, and CAN, Yin *et al.* revealed that the expression of Gas6 in allografts increased, along with the increased expression of Axl in acute tubular necrosis [109]. In a podocyte ablation model of glomerular albuminuria, the elevated expression of Gas6/Axl was involved in a protective tubular proliferative response [110].

Akt signaling is the main downstream effector of Gas6/Axl, and Gas6/Axl also modulates Wnt, VEGF, Erk1/2 and integrin-mediated signaling [103]. In endothelial cell, Gas6/Axl was decreased by HG by influencing Akt signaling (not MAPK signaling), which resulted in changes of adhesion molecules and VEGF/VEGFR2 expression, leading to the regulation of endothelial adhesion and angiogenic functions [106]. Warfarin, which inactivates Gas6, can inhibit mesangial and glomerular hypertrophy and the increase in albuminuria in STZ-rats [4]. In NS and DN, induced by STZ, loss of Gas6 prevented mesangial cell proliferation, glomerular hypertrophy and sclerosis, resulting in proteinuria and survival improvement [111]. In conclusion, Gas6/Axl plays a critical role in the progression of various kidney diseases. Blocking the Gas6/Axl pathway may be a potent therapeutic target for CKD.

## 10. Tyrosine Kinase Inhibitors

Of note, tyrosine kinase Inhibitors (TKIs) are already known as the first-line therapies for various malignancies. Many basic and clinical studies have also suggested the potential anti-fibrotic effects of TKIs in a variety of tissues. For example, in skin, lung, liver, systemic sclerosis and renal fibrosis models, the PDGFR inhibitor (imatinib) could inhibit fibroblast activation, reduce ECM synthesis, prevent fibrosis formation and improve the formed fibrosis [12]. In the kidney, imatinib has also been shown to attenuate pathological changes in different models of CKD, including experimental glomerulonephritis [112,113], DN [114], LN [115], and CAN [116]. In addition, AG1296, a specific TKI for FGFR, also shows the inhibitory effect on FGF-2-induced renal fibroblast proliferation [56]. In addition, Erlotinib, a TKI for EGFR, can significantly decrease TGF-β-mediated fibrogenesis [64]. In a rat model of hyperuricemic nephropathy, administration of gefitinib, a highly selective EGFR inhibitor, could inhibit activation of renal interstitial fibroblasts and expression of ECM proteins, leading to improving renal dysfunction and urine microalbumin [117].

Since animal studies have demonstrated that renal fibrosis is a process involved in the activation of multiple RTKs, administration of a combined therapy with several RTK inhibitors (or an inhibitor with the capacity to block multiple RTKs) may result in a better anti-fibrotic effect. Indeed, our recent studies have shown that treatment with suramin, an inhibitor of multiple RTKs, can dramatically inhibit development and progression of renal fibrosis [16,118] and attenuate glomerular and vascular injury while reducing inflammatory responses [16]. In addition, suramin was effective in attenuating inflammation and fibrosis while improving renal function in DN [119,120]. The recent studies on the antifibrotic effects of TKIs in renal disease are listed in Table 2.

Considered as a multiple TKIs, nintedanib (BIBF1120) is a potent, oral, small-molecule agent, which can concurrently block PDGFR-α and -β, VEGFR1, 2 and 3, FGFR1, 2 and 3, Src and the PLT-3 family RTKs [121]. It can competitively bind to ATP box in targeted RTKs and inhibit self-phosphorylation of those RTKs, resulting in attenuation of downstream signaling pathways [122]. In an animal model of pulmonary fibrosis, nintedanib was shown to induce pro-MMP-2 expression, inhibit matrix expression of protease tissue inhibitor-2 (TIMP-2) in lung fibroblasts [123], reduce transdifferentiation of fibroblasts to myofibroblastsinduced by TGF-β, and reduce collagen synthesis [124]. In clinical trials, nintedanib was shown to effectively improve lung function in patients with idiopathic pulmonary fibrosis (IPF), reduce acute attack frequency and improve life quality [125]. Nintedanib has been approved by the Food and Drug Administration (FDA) for clinical applications of IPF [126,127,128]. To date, there are no reports on the anti-fibrotic effect of nintedanib in the kidney, our recent studies have showed that it is also a potent agent that inhibits renal fibroblast activation and renal fibrogenesis (personal communication).

Fibrosis is the common and ultimate stage of organ fibrogenesis due to a variety of different etiologies or pro-fibrotic factors. Once initiated, a variety of pro-fibrotic pathways involved in and influence each other, leading to fibrosis progress continuing to be persistent and irreversible, even if one pathway to be suppressed. Therefore, it suggests that less selective, smaller molecules, and multiple-receptors inhibitors that influence several pathways are more effective in inhibiting tissue fibrosis. From the process of basic research to clinical application of nitendanib on IPF, it is suggested that multiple-receptor TKIs may not only show a higher effectiveness but also necessitate more careful clinical evaluations in regard to their underlying side effects for the treatment of renal fibrosis.

It should be noted that TKIs can cause some side effects. The most common side effects include edema, fatigue, nausea and vomiting, diarrhea, generalized rash, new onset proteinuria, and muscle weakness [129]. There have also been reports showing renal complications in the malignant patient after treatment with gefitinib, including minimal change, nephritic syndrome [130], tubulointerstitial nephritis and IgA glomerulonephritis [131,132]. However, the renal complications may be secondary to the original tumors. Therefore, the efficacy and tolerability of TKIs in treating fibrotic disease should be considered in clinical practice.

## 11. Conclusions

Receptor tyrosine kinase signaling plays a key role in a wide variety of renal cellular processes. There is substantial evidence from *in vitro* studies and animal models that support specific receptor tyrosine kinase involvement in the pathogenesis of renal fibrosis. A large number of animal studies have suggested that tyrosine kinase inhibitors have the potential value in slowing renal fibrosis progression. Further, basic research is needed to elucidate how specific kinases contribute to the pathogenic processes in renal fibrosis. Moreover, selection of appropriate tyrosine kinase inhibitors, rigorous preclinical investigations, and large randomized-controlled clinical trials are necessary for the prospect of tyrosine kinase inhibitors therapy for renal fibrosis to ensure more beneficial and less adverse effects.

## Figures and Tables

**Table 1:** Classification of receptor tyrosine kinases (RTKs) superfamilies and ligands.

RTK Superfamily	Members	Ligands
EGFR	EGFR (Erb1/Her1), Erb2/Her2, Erb3/Her3, Erb4/Her4	TGF-α, EGF, HB-EGF, amphiregulin, betacellulin, epiregualtin, epigen
PDGFR	PDGFR-αα, PDGFR-αβ, PDGFR-ββ, M-CSFR, SCFR, FLT3L	PDGF-A, -B, -C, -D
VEGFR	VEGFR1 (Flt-1), VEGFR2 (KDR), VEGFR3 (Flt-4)	VEGF-A, -B, -C, -D, -E
FGFR	FGFR1, FGFR2, FGFR3, FGFR4	FGF1, FGF2
IGFR	Type I IGFR	IGF-1, IGF-2
DDR	DDR1, DDR2	collagen I–VI and VIII
Axl	Axl, MER, TYRO3	Gas6, protein S
STYK	STYK1	STYK
CCK	CCK4	CCK, gastrin
NGFR	TRKA, TRKB, TRKC	NGF, BDNF, NT3, NT4
HGFR	MET, RON	HGF, MSP
EPHR	EphA1-8, EphA10, EphB1-4, EphB6	ephrin-A1-5, ephrin-B1-3
TIE	TIE1, TIE2	angiopoietin-1-4
RYK	RYK	RYK
RET	RET	GDNF
ROS	ROS	orphan
LTK	LTK, ALK	Orphan/Pleiotrophin
ROR	ROR1, ROR2	Wnt5A
MUSK	MUSK	Agrin, Dok-7
LMR	LMR1, 2, 3	(vestigial ECD)

EGFR: Epidermal growth factor receptor; Erb: estrogen receptor beta; Her: Human epidermal growth factor receptor; TGF: Transforming growth factor; EGF: Epidermal growth factor; HB-EGF: heparin-binding epidermal growth factor-like growth factor; PDGFR: Platelet-derived growth factor receptor; M-CSFR: Macrophage colony-stimulating factor receptor; SCFR: Stem cell factor receptor; FLT3L: Fms-like tyrosine kinase 3 ligand; PDGF: Platelet-derived growth factor; VEGFR: Vascular endothelial growth factor receptor; VEGF: Vascular endothelial growth factor; PlGF: Placental growth factor; FGFR: Fibroblast growth factor receptor; FGF: Fibroblast growth factor; IGFR: Insulin-like growth factor receptor; IGF: Insulin-like growth factor; DDR: Discoidin domain receptor; Axl: From the Greekword anex-elekto, or uncontrolled, aTyro3 protein tyrosine kinase; MER: Membrane bound oestrogen receptor; Gas: Growth arrest-specific gene; STYK: Serine/threonine/tyrosine kinase; CCK: Colon carcinoma kinase; NGFR: Nerve growth factor receptor; TRK: Tyrosine kinase receptor; NGF: Nerve growth factor; BDGF: Brain-derived neurotrophin factor; NT: Neurotrophin; HGFR: Hepatocyte growth factor receptor; MET: Mesenchymal-epithelial transition factor; RON: Recepteur d’Origine nantais; HGF: Hepatocyte growth factor; MSP: Macrophage stimulating protein; EPHR: Ephrin receptor; TIE: Tyrosine kinase receptor in endothelial cells; RYK: Receptor related to tyrosine kinases; RET: Rearranged during transfection; GDNF: Glial cell line-derived neurotrophic factor; ROS: RPTK expressed in some epithelial cell types; LTK: Leukocyte tyrosine kinase; ALK: Anaplastic lymphoma kinase; ROR: Receptor orphan; MuSK: Muscle-specific kinase; Dok: Downstream-of-kinase; LMR: Lemur; ECD: Extracellular domain.

**Table 2:** Effects of TKIs in animal models of renal disease.

Animal Models	Intervention	Targets	Effects	Reference
Murine unilateral ureteral obstruction	Suramin	PDGFR, EGFR	Attenuates glomerular and vascular injury and reduces inflammatory responses	[22]
Rat chronic anti-Thy 1.1 glomerulonephritis	B-specific oligonucleotideaptamer	PDGFR	Decreases proteinuria and improves renal function; Inhibits glomerulosclerosis and tubulointerstitial fibrosis	[23]
Rat chronic anti-Thy 1.1 glomerulonephritis	Neutralizing anti-PDGF-D IgG	PDGFR	Decreases proteinuria and improves renal function; Inhibits glomerulosclerosis and tubulointerstitial fibrosis and EMT	[24]
Rat acute anti–Thy 1.1 glomerulonephritis	Trapidil	PDGFR	Inhibits mesangial cells proliferation and matrix accumulation	[25]
Rat ischemia/reperfusion injury	Trapidil	PDGFR	Increases serum creatinine and mortality rate; Inhibits proliferation of tubular epithelial cells	[26]
Rat acute anti-Thy 1.1 GN	Imatinib	PDGFR	Inhibits mesangial cells proliferation and matrix accumulation	[16,122,123]
Murine streptozotocin-induced diabetes	Imatinib	PDGFR	Decreases albuminuria, glomerular and tubulointerstitial damage	[16]
Murine lupus	Imatinib	PDGFR	Improves survival, decreases proteinuria, glomerular and tubulointerstitial damage	[16]
Rat unilateral ureter obstruction	AG 1295	PDGFR	Inhibits tubulointerstitial fibrosis	[27]
Rat crescentic glomerulonephritis	sFlt-1	VEGFR	Accelerates proteinuria with massive ascites, glomerulosclerosis, and interstitial fibrosis	[51]
Rat model of 5/6 nephrectomy	Suramin	PDGFR,EGFR	Inhibits glomerulosclerosis and vascular sclerosis, as well as inflammation	[82]
Murine model of unilateral I/R model	Suramin	PDGFR,EGFR	Decreases tubular cell apoptosis, dedifferentiation and proliferation	[83]
Rat model of hyperuricemic nephropathy	Gefitinib	EGFR	Prevented renal dysfunction, reduced urine microalbumin, and inhibited activation of renal interstitial fibroblasts and expression of extracellular proteins	[127]
